# Point-of-Care Testing for G6PD Deficiency: Opportunities for Screening

**DOI:** 10.3390/ijns4040034

**Published:** 2018-11-19

**Authors:** Athena Anderle, Germana Bancone, Gonzalo J. Domingo, Emily Gerth-Guyette, Sampa Pal, Ari W. Satyagraha

**Affiliations:** 1 PATH, 2201 Westlake Ave, Suite 200, Seattle, WA 98121, USA; 2 Shoklo Malaria Research Unit, Mahidol–Oxford Tropical Medicine Research Unit, Faculty of Tropical Medicine, Mahidol University, 68/30 Bantung Road, PO Box 46 Mae Sot, Tak 63110, Thailand; 3 Centre for Tropical Medicine and Global Health, Nuffield Department of Medicine, University of Oxford, Old Road campus, Roosevelt Drive, Oxford OX3 7FZ, UK; 4 Eijkman Institute, Jalan Diponegoro 69, Jakarta 10430, Indonesia

**Keywords:** glucose-6-phosphate dehydrogenase, G6PD deficiency, point-of-care, diagnostics, malaria, *Plasmodium vivax*

## Abstract

Glucose-6-phosphate dehydrogenase (G6PD) deficiency, an X-linked genetic disorder, is associated with increased risk of jaundice and kernicterus at birth. G6PD deficiency can manifest later in life as severe hemolysis, when the individual is exposed to oxidative agents that range from foods such as fava beans, to diseases such as typhoid, to medications such as dapsone, to the curative drugs for *Plasmodium* (*P.*) *vivax* malaria, primaquine and tafenoquine. While routine testing at birth for G6PD deficiency is recommended by the World Health Organization for populations with greater than 5% prevalence of G6PD deficiency and to inform *P. vivax* case management using primaquine, testing coverage is extremely low. Test coverage is low due to the need to prioritize newborn interventions and the complexity of currently available G6PD tests, especially those used to inform malaria case management. More affordable, accurate, point-of-care (POC) tests for G6PD deficiency are emerging that create an opportunity to extend testing to populations that do not have access to high throughput screening services. Some of these tests are quantitative, which provides an opportunity to address the gender disparity created by the currently available POC qualitative tests that misclassify females with intermediate G6PD activity as normal. In populations where the epidemiology for G6PD deficiency and *P. vivax* overlap, screening for G6PD deficiency at birth to inform care of the newborn can also be used to inform malaria case management over their lifetime.

## 1. Introduction

Glucose-6-phosphate dehydrogenase (G6PD) deficiency is one of the most common X-linked genetic blood disorders in the world, impacting more than 400 million people. Individuals that are G6PD deficient can develop severe jaundice in the neonatal period and acute hemolytic anemia when exposed to certain infections and drugs or when ingesting certain foods such as fava beans [1,2].

Females carry two copies of the G6PD gene, such that they can be homozygous for normal alleles (*g6pd*_norm/norm_), homozygous for deficient alleles (*g6pd*_def/def_) or heterozygous with one deficient and one normal G6PD allele (*g6pd*_def/norm_). Wild-type homozygous females will present phenotypically as normal, with a G6PD activity level greater than 80% of the normal activity level, and homozygous females with two deficient alleles will present with a G6PD activity level less than 30%. However, heterozygous females with a deficient and a normal allele have a much broader phenotype, which lies mostly in the intermediate 20–80% G6PD activity ranges. With a single allele, G6PD activity levels in males are either normal or deficient [3,4,5,6,7].

G6PD deficiency is relevant to newborns because of the higher risk neonates with G6PD deficiency face in developing non-physiologic hyperbilirubinemia. Elevated levels of serum bilirubin (SBR) can pass the blood-brain barrier and lead to a range of neurologic disorders, including acute bilirubin-induced encephalopathy, kernicterus (chronic neurologic disease), and even death [8,9,10]. In term and late preterm newborns (≥35 weeks of gestational age), hyperbilirubinemia can be treated with blue-light phototherapy and, in the most severe cases, with exchange transfusion following universally-accepted guidelines based on an age-specific SBR nomogram.

Severe hyperbilirubinemia usually develops within one week of birth but can also develop at a later stage. A hospital or birthing center’s ability to monitor SBR levels is crucial for clinical management. In places where SBR testing is not routine, the ability to identify risk factors of hyperbilirubinemia before hospital discharge (ideally at birth) should prompt SBR testing and, together with parents and health workers, instigate education about signs of hyperbilirubinemia and guide the follow-up at home. For newborns with G6PD deficiency in particular, clinical management both in the hospital and at home includes an avoidance of hemolytic triggers (including drugs, food, and other substances). In 1989, the World Health Organization (WHO) working group on G6PD deficiency recommended that “whenever possible, neonatal screening should be performed … in populations where G6PD deficiency is common (i.e., where it affects more than three to five percent of males)” [11].

Geographically, G6PD deficiency is ethnically constrained, resulting in significant variability in risk, even within limited geographical boundaries [12,13]. Overall, populations with historic or current exposure to malaria typically have higher prevalence for G6PD deficiency with a mean prevalence of approximately 8.0% in malaria-endemic countries [12]. There is data to suggest that G6PD deficiency—while not protective against red blood cell invasion from parasites—is protective against severe clinical forms of malaria, which may explain the epidemiological overlap between G6PD deficiency and malaria [14,15,16,17].

In 2016, there were an estimated 8.5 million *Plasmodium* (*P.*) *vivax* cases, representing more than 35% of malaria cases outside of Africa [18]. As countries transition from malaria control to elimination, the predominant form of malaria often also transitions from *P. falciparum* to *P. vivax*; *P. vivax* accounts for 70% of malaria cases in countries with fewer than 5000 cases per year [19]. Case management of *P. vivax* (and *P. ovale*) is complicated compared to that of *P. falciparum*, due to the ability of *P. vivax* parasites to reside dormant in the liver as hypnozoites [20,21]. Hypnozoites are not susceptible to typical antimalarial drugs, which target the blood forms of the parasite, and can therefore cause relapse of the disease, weeks or months after primary infection. Relapse infections are a major source of disease burden in *P. vivax*–endemic populations [22,23]. The only class of antimalarial drugs that can cure individuals of *P. vivax* malaria is 8-aminoquinoline drugs; however, they can cause severe hemolysis in patients with G6PD deficiency. Historically, a high-dose, 14-day regimen of primaquine has been used for a radical cure of patients with *P. vivax*. Recently, tafenoquine, under the brand name of Krintafel, was approved by the Food and Drug Administration as a single-dose regimen to treat patients with confirmed *P. vivax* infection. WHO recommends testing for G6PD deficiency before administration of primaquine [24], and given the toxicity profile of the single-dose regime, testing will be required prior to administration of tafenoquine.

Due to an increased awareness of the morbidity caused by *P. vivax* relapse, the contribution of *P. vivax* relapse to onward malaria transmission, the commitment to malaria elimination in many predominantly *P. vivax*–endemic countries and the imminent availability of tafenoquine, there is renewed focus to address relapsed *P. vivax* infections by increasing access to a radical cure. In response, diagnostics manufacturers have advanced the development of point-of-care tests for G6PD deficiency that will be required for use in malaria case management.

This review discusses the overlap between screening for G6PD deficiency in newborns and testing for G6PD deficiency to inform malaria case management as well as the availability of new technologies that can bring G6PD testing to underserved and remote populations where G6PD deficiency and *P. vivax* malaria predominates.

## 2. Testing for G6PD Deficiency

The G6PD deficiency status of an individual can be characterized by genotype or by phenotype (Table 1). There are increasingly effective tools for G6PD genotyping both in terms of cost and timeliness [25]. For males, the genotype is sufficient to unambiguously assign a phenotype. For females, the genotype of females heterozygous for a G6PD normal and a G6PD deficient allele cannot be unambiguously phenotypically classified as their blood enzyme activities can range between 20–80% of a normal value, with the majority close to the 50% normal activity range. Regardless, it can be anticipated that G6PD genotyping will increase in screening programs through next-generation sequencing assays [26,27,28].

The G6PD phenotype is primarily described in terms of G6PD activity normalized for hemoglobin or red blood cell count. It has been challenging to define a single universal normal (100%) G6PD activity value, so that classification of the G6PD status of an individual is defined as the percentage of a normal value determined locally. G6PD phenotype classifications for purposes of test performance evaluation were recently described by the WHO [29]. Males with less than 30% activity are considered as deficient and males with greater than 30% activity should be considered as normal [29]. Females with less than 30%, 30–80%, and greater than 80% G6PD activity are considered G6PD deficient, intermediate, and normal, respectively [29]. Another way of defining the phenotype is by cytochemistry, wherein individual red blood cells are labeled for G6PD activity levels, and then, typically either by eye (if by microscopy) or by gating (if by flow cytometry), cells are dichotomously classified as deficient or normal and the ratio of the two can then inform a phenotypic classification (Table 1). While extremely informative, this latter approach is primarily used as a research tool and will not be further described here. From a clinical perspective, it is the G6PD phenotype that informs the risk of someone developing G6PD deficiency–related pathologies.

The biochemical assays that measure enzyme activity include two categories of G6PD tests, qualitative and quantitative. By convention, a G6PD deficient individual is considered a true positive, and a G6PD normal individual a true negative, such that sensitivity refers to the ability of a test to identify all true G6PD deficient individuals and specificity is the ability of the test to identify all true G6PD normals. A quantitative test is used as the reference standard [29,30]. The qualitative tests can only really discriminate G6PD deficient individuals from intermediate and normal individuals, and as such, heterozygous females with G6PD activity 30–40% of normal are typically classified as normal even though they have very low G6PD activity levels [30,31]. The qualitative tests have a discriminatory threshold for deficient and normal at the 30–40% activity level and can display good sensitivity for deficient males, and females with two G6PD deficient alleles, as they typically have G6PD activity below 30% normal. If there is a need to differentiate heterozygous females with low intermediate activity levels (40–50%) from G6PD normal individuals, or in other words raise the threshold G6PD activity level, the sensitivity of the qualitative test then begins to drop [31]. However, with a quantitative test, as long as there is good correlation with the reference assay, and with a gradient close to unity, the sensitivity can be kept high along the whole dynamic G6PD activity range. The most widely used qualitative test and the clinical standard of care in most hospitals is the fluorescent spot test (FST), which consists of observing nicotinamide adenine dinucleotide phosphate (NADPH) production under a long wave ultraviolet light source [32]. In newborns, the above-described limitation of the qualitative test combined with the high reticulocyte counts typically leads to a misdiagnosis of females with low G6PD activity levels at risk of developing G6PD-associated complications as normals. In other words: the sensitivity drops. There is an increasing recognition that the thresholds for defining newborns at risk of G6PD-associated complications need to be higher than the discriminatory cutoffs used by qualitative tests such as the FST [33,34,35,36].

In the context of G6PD screening, the most common approach has been to include G6PD screening within other screening programs that are typically congenital hypothyroidism screening. In these programs, the specimen source is often the heel stick (in some cases, cord blood), stored and transferred via dried blood spots (DBS), commonly known as the Guthrie card. Samples are assessed primarily via qualitative or quantitative biochemical methods, although genotyping is also performed. Screening typically utilizes high throughput instrumentation. Several strong external quality assurance systems have been put in place to assure the quality of these large volume testing facilities [37,38].

## 3. Newborn Screening Practices for G6PD Deficiency

A recent review focusing on G6PD deficiency testing within newborn screening (NBS) practices highlights a heterogeneity in practices that are not directly correlated to the prevalence of G6PD deficiency within a country [41]. Africa and the Middle East present the highest prevalence of G6PD deficiency; however, these regions have the lowest coverage of newborn screening for G6PD deficiency. Newborn screening coverage for G6PD deficiency is the highest in the Asia Pacific region, with at least six countries providing full coverage and several also providing this service to sub-populations or access to private-sector services [41]. Additionally, in many countries that conduct newborn screening for G6PD deficiency, screening is primarily accessible to urban populations near facilities that provide the service.

Expert guidance from neonatologists has outlined key considerations when deciding whether or not to adopt or scale newborn screening for G6PD deficiency [42]. Key questions are: (1) whether testing should take place before babies leave the hospital, (2) whether screening should be universal or targeted toward babies at greatest risk and (3) what screening method should be used [43]. Additional considerations include the cost-effectiveness of screening, the frequency and severity of G6PD deficiency in a specific population, availability and efficacy of appropriate diagnostics options, and the capacity of the health system to provide appropriate counseling to parents and providers [44,45]. Section 3.1 and Section 3.2 below describe practices in some countries in the context of these considerations and are by no means comprehensive.

### 3.1. Newborn Screening for G6PD Deficiency in the United States and Europe

In the United States, screening for G6PD deficiency is only routinely done through the newborn screening programs in two states: Pennsylvania and DC [41,46,47]. Facilities outside of those states may choose to adopt universal or targeted screening practices independently [41,47]. In Europe, newborn screening guidelines vary widely with little consensus on what should be included. Greece is the only country with nationwide coverage, while Italy has partial coverage and other countries have targeted programs [41,48]. However, in both the United States and Europe, migration makes it increasingly complicated to predict the prevalence of G6PD deficiency, the specific genotype, and the risk that certain newborn complications are related to G6PD status [49].

The American Academy of Pediatrics recommends that newborns with jaundice are screened for G6PD deficiency when family history or background suggests a likelihood of G6PD deficiency or when the response to phototherapy is poor [50,51,52]. Multiple methods are used for newborn screening with varying performance [53,54]. In the United States, some reports have concluded that fluorescent spot test (FST) methods are sufficient, while others indicate they are inadequate, particularly for females, due to the lack of an accurate quantitative measurement [55,56,57,58].

There is some concern that among clinicians practicing in the United States, the prevalence and clinical implications of G6PD deficiency are underappreciated [59]. Nonetheless, it is evident that American clinicians support newborn screening for G6PD [60,61,62]. There is some evidence to suggest that hospital-based G6PD deficiency screening is feasible and that, when paired with parental education around risk factors and triggers, the negative health impacts from hyperbilirubinemia are limited [9,47,63]. In Greece, an assessment of the national screening program from 1977–1989 was deemed justified in areas of high G6PD prevalence [64,65]. Similarly, a robust G6PD newborn screening program paired with health education programs implemented in the Sassari district of Sardinia, Italy, was associated with a 75% decline in clinical complications associated with G6PD deficiency. Notably, this decline was disproportionally observed among boys, suggesting that the intervention is less effective in girls, possibly driven by inadequacies in the screening method for female populations [66].

### 3.2. Newborn Screening for G6PD Deficiency in the Asia Pacific

The prevalence of G6PD deficiency is close to or more than 5% throughout the Asia Pacific region, but many countries do not have NBS programs, and for countries that do, the programs are often inefficient, with many excluding screening for G6PD deficiency. Countries of the Greater Mekong subregion where G6PD deficiency is high (Thailand, Myanmar, Laos, Vietnam and Cambodia) currently have no NBS programs despite evidence indicating a need otherwise [67].

For example, in Thailand, several studies have shown a high prevalence of G6PD deficiency [68,69] as well as an association of the deficiency with neonatal hyperbilirubinemia; however, a national NBS program for G6PD deficiency has not been set up [70,71,72]. Similarly, an NBS program for G6PD deficiency in Indonesia has not been implemented; a few private hospitals may screen for G6PD deficiency in newborns, while others will screen for G6PD deficiency only when there is an indication of non-physiological jaundice. While NBS in Indonesia started in 1999, it was only for congenital hypothyroidism using a heel stick sample, and coverage is <1% despite this being a national program [73], indicating a large systemic obstacle that would need to be addressed prior to G6PD deficiency screening implementation.

In the Philippines, where the prevalence of G6PD deficiency ranges from 4.5% to 25.7%, testing for G6PD deficiency is included in its newborn screening program, which is carried out within 24 h of birth; however, coverage remains low at 28% [74]. Both the Philippines and Taiwan implemented national NBS programs in 1998 and 1987, respectively. These programs utilize FST and have developed follow-up systems for G6PD-deficient individuals to receive follow-up confirmatory testing using spectrophotometry. In Taiwan, the follow-up system also includes medical care and genetic counseling [67].

Examples of successful G6PD deficiency newborn screening are Malaysia and Singapore. Malaysia has been conducting G6PD NBS since the 1970s from cord blood, and the coverage is >95% in the population and funded by the government [67]. Similarly, Singapore recognized the important role of G6PD deficiency in kernicterus and started NBS for G6PD deficiency in 1965 using cord blood as well [75]. The reported nationwide coverage for NBS is >99%. The government subsidized about 40–60% of the cost of NBS within public hospitals and has since eradicated kernicterus due to G6PD deficiency [67]. The current policy is to keep G6PD-deficient babies longer in hospitals to avoid hemolytic triggers from the environment [76].

## 4. G6PD Testing for Malaria Case Management

**Figure 1:**
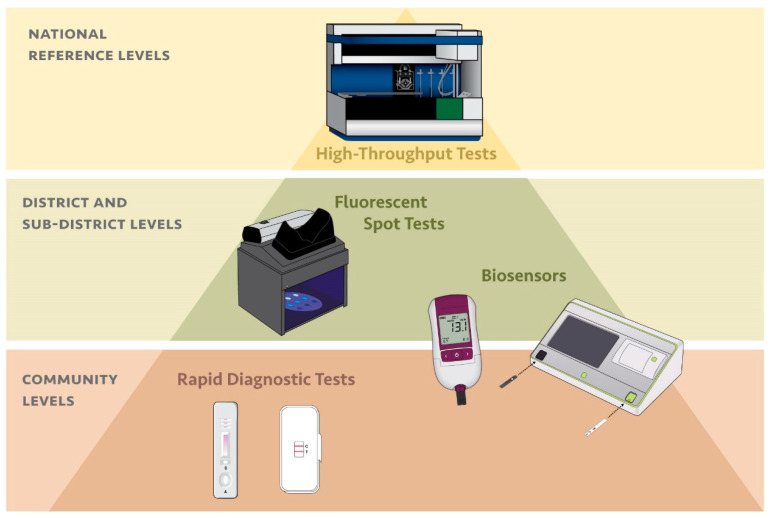
Alignment between diagnostic platform for G6PD deficiency and tier of health care facility based on complexity of the diagnostic test and the typical resources available at each type of facility.

In contrast, G6PD testing to inform malaria case management using primaquine has typically not been possible due to the complexity of current G6PD test methods, which are not compatible with the remote and under-resourced clinical and laboratory settings where a majority of malaria patients seek care (Figure 1). In recent years, there has been an increase in the availability of point-of-care tests for G6PD deficiency. The CareStart G6PD (Access Bio, Somerset, NJ, USA) rapid diagnostic test is perhaps most aligned with these clinical settings, however, as a qualitative test it has some inherent limitations compared to quantitative tests (Table 2) [77,78,79]. The SD Biosensor STANDARD G6PD test (Suwon, Korea) represents a new point-of-care product that brings quantitative G6PD measurement normalized for hemoglobin capabilities to lower-tier clinical and laboratory settings [80]. Several other point-of-care tests for G6PD deficiency are also in development on different platforms, such as the Access Bio CareStart G6PD Biosensor (Somerset, NJ, USA) and the FINDER platform from Baebies (Durham, NC, USA) [56,81]. The benefit of the instrumented quantitative products is that they can address the inherent enzyme temperature variance through temperature correction, sustaining their accuracy and therefore utility over a broader temperature range, in contrast to the qualitative tests. More critically, just as for neonatal screening, quantitative testing is increasingly relevant for providing equal access to both males and females to both high-dose primaquine and the recently FDA- and TGA-cleared antimalarial drug tafenoquine [39]. However, there is an added level of complexity and cost to requiring an instrument to run G6PD tests in each facility where G6PD testing may be required.

## 5. New Opportunities for G6PD Screening: Synergies and Considerations

The advent of these new G6PD testing technologies raises new opportunities to address inequity in access to newborn screening in countries with high G6PD deficiency prevalence. Additionally, the overlap in epidemiology for G6PD deficiency and *P. vivax* provides a synergistic need for testing that may warrant supporting the intervention. Yet, there are several considerations that should be taken into account when assessing what technology should be included when testing is implemented.

### 5.1. Overlap in Desired Product Characteristics

Point-of-care G6PD tests are designed to provide fast turnaround, typically within ten minutes. This is an essential characteristic for malaria case management because patients are lost to follow-up if they are asked to return for their G6PD test result several days later. Fast turnaround is also essential for neonatal clinics in low-resource settings, as the mother and child rarely stay in the hospital longer than 24 h and systems for remote testing and test result return are highly inefficient. A limitation is that the throughput required for newborn testing is likely to be significantly higher than that for malaria testing in some settings. Additionally, the time it takes for high throughput tests to provide results may not be quick enough to inform the care of sick newborns, making a point-of-care test that can provide results within ten minutes a preferred option, even when routine screening methods are available.

### 5.2. Work Flow and Sample Type

The point-of-care tests that are on the market have been designed and validated for use with fresh whole blood specimens, with or without anticoagulant; however, they have not been shown to be compatible with dried blood spots, the primary specimen used in newborn screening programs. Alternatively, operations research would need to assess the feasibility of incorporating the point-of-care tests, as is, into the current workflow in delivery wards using capillary samples directly from the heel stick. In some contexts, validating the products with cord blood may be useful.

### 5.3. External Quality Assurance

Newborn screening programs have significantly invested in quality assurance systems, which are compatible with high throughput testing facilities but are not compatible with more decentralized lower throughput testing facilities. Quality control reagents formulated to support high throughput facilities can be amortized over many samples, which would not be the same for lower throughput facilities, resulting in significantly increasing the price of testing. Pragmatic solutions that address these differences, such as new formulations for control reagent presentation, will need to be thought out and tested.

### 5.4. Record Keeping

The return-on-investment or value proposition for G6PD testing at birth is highly dependent on the reliability of diagnosis done at birth and the ability of the test result to stay with the individual and individual’s caretakers, which minimizes the need to retest the individual later in life. Record keeping can be very challenging in many malaria-endemic settings, which means setting- and population-specific solutions are often required.

### 5.5. Awareness and Sensitization

Record keeping is key for determining value proposition but is only valuable itself if the parties involved are sensitized to the implications of G6PD deficiency. This would include understanding how to prevent exposure to triggers for hyperbilirubinemia and hemolysis in G6PD-deficient individuals as well as how to identify and react according to the early onset of associated symptoms.

### 5.6. Cost-Effectiveness

In many low-resource settings, the priority for incorporating G6PD deficiency testing over other interventions will be hard to justify in the context of the many competing health system limitations and priorities. A series of factors will contribute to the overall value proposition in those settings, which include the prevalence of G6PD deficiency in the local population served, the likelihood that a G6PD-deficient individual will suffer associated pathologies later in life and the ability for the test result at birth to be associated with the individual throughout their life. Cost-effectiveness assessments that focus only on the short-term benefits of G6PD testing at birth are unlikely to support prioritizing the intervention. A framework for assessing the cost-effectiveness for G6PD testing at birth in settings with high prevalence of G6PD deficiency and other triggers for G6PD deficiency–associated hemolysis should be considered. These triggers can include antibiotics, certain foods, and several medications. In populations where there is also a high prevalence of *P. vivax* malaria, and radical treatment with 8-aminoquinolines is provided, the significant health benefit of preventing malaria relapse versus the costs of hospitalization of patients reacting to the drugs should be included. Cost-effectiveness models for G6PD testing for a radical cure have been developed; however, these do not integrate the use of tests at birth to avoid complications, or beyond that, of malaria case management.

## 6. Summary

New quantitative point-of-care technologies that address both the need for immediate results to mitigate the risk of hyperbilirubinemia and the need to provide reliable and actionable results for management of newborns or patient treatment decisions may help spur stronger and more comprehensive newborn screening efforts for G6PD deficiency in settings that do not have practical access to centralized screening programs. Testing must be accompanied with community awareness of and sensitization to G6PD deficiency along with robust record keeping such that the investments are maximized beyond the first days of life. In malaria-endemic regions, G6PD testing will provide access to the best standard of care, which is a radical cure of *P. vivax* malaria. Operations research is required to assess the feasibility and effectiveness of G6PD testing with these new point-of-care tests at birth.

## Figures and Tables

**Table 1:** Association between genotype and phenotype. Two methods of measuring phenotype are shown: (1) by cytochemical staining, where red blood cells (RBC) are arbitrarily assigned as having high glucose-6-phosphate dehydrogenase (G6PD) activity or low G6PD activity [5] and (2) by spectrophotometric G6PD enzyme activity measurement in whole blood. The activity is described in terms of percentage of a population’s normal value [39,40].

Genotype		Phenotype	
		% RBC with High G6PD Activity (Cytometry)	% Normal G6PD Activity (Spectrophotometry)
**Males**			
hemizygous normal	(+)	>85%	>30%
hemizygous deficient	(−)	<10%	≤30%
**Females**			
homozygous normal	(+_1_/+_1_)	>85%	>70%
heterozygous normal	(+_1_/+_2_)		
heterozygous normal/deficient	(+/−)	10–85%	~20–80%
heterozygous deficient	(−_1_/−_2_)	<10%	≤30%
homozygous deficient	(−_1_/−_1_)		

**Table 2:** Characteristics of qualitative and quantitative point-of-care G6PD tests.

Qualitative	Quantitative
Accurately classifies males	Accurately classifies males
Females with intermediate G6PD activity classified as normal	Accurately classifies females
Does not require an instrument	Requires an instrument
Cannot correct for operating temperature, typically resulting in a more limited operating temperature range	Corrects for temperature allowing for a broader operating temperature range
Time-to-result < 10 min	Time-to-result < 10 min
Low to moderate complexity	Moderate complexity
